# Is the African Vaccine Manufacturing Accelerator a decoupling mechanism?

**DOI:** 10.1093/inthealth/ihae032

**Published:** 2024-05-07

**Authors:** Abiodun E Awosusi

**Affiliations:** Doctoral Student, Ivey Business School, Western University, 1255 Western Road, London, Ontario, Canada N6G 0N1; Affiliate Trainee at the Centre for Research on Health Equity and Social Inclusion c/o Innovation Works 201 King Street, London, Ontario, Canada N6A 1C9

**Keywords:** decoupling, equitable access, global health, knowledge transfers, partnerships, regulatory system, vaccine manufacturing, vaccines

## Abstract

This article explores how the African Vaccine Manufacturing Accelerator can support the sustainable production of vaccines in Africa. It highlights the value of the accelerator in relation to the Regional Vaccine Manufacturing Collaborative. The author proposes that this novel financing instrument should be well-designed and implemented in line with the targets of the Partnerships for African Vaccine Manufacturing. It should not be a decoupling tool to appease the institutional environment of the global vaccine market, but a sustainable demonstration of the goodwill and commitment of political and technical leaders to ensure equitable access to routine and epidemic-related vaccines in Africa.

GAVI, the Vaccine Alliance aims to support regional production of vaccines through technical and financial mechanisms including the African Vaccine Manufacturing Accelerator (AVMA).^1^ This aligns with GAVI's regional manufacturing strategy and the ideals of the Regionalized Vaccine Manufacturing Collaborative (RVMC)^2^ co-developed by the World Economic Forum, the National Academy of Medicine and the Coalition for Epidemic Preparedness Innovations: the collaborative recommends an eight-pillar framework to facilitate the development of a sustainable regional vaccine value chain. These steps require structural and ancillary changes within the global health ecosystem as countries build national and regional capacities.^3^

The AVMA is envisioned to provide a set of performance-based incentives to facilitate vaccine production in Africa and enable African manufacturers to gain access to the global vaccines market. Eligible manufacturers can receive milestone fund and accelerator payments when they meet regulatory and competitive tender targets, respectively.^1^ These contingent payments are expected to offset the high costs of meeting regulatory standards and reaching competitive production scale for priority vaccines on the continent. Some of the priority vaccines target yellow fever, malaria, cholera, Ebola, measles–rubella and rotavirus. Since its approval by the GAVI Board, the AVMA has been well received by multiple stakeholders committed to expanding vaccine production capacity in Africa, with high expectations for its adaptive implementation and eventual success.

Achieving prequalification by the World Health Organization (WHO) is a rate-limiting step for African manufacturers to receive milestone and ancillary payments through the AVMA.^1^ This signals to African stakeholders and development partners to invest in regulatory system strengthening so that African manufacturers can achieve regulatory and quality milestones required for export demand. Although their scope of products differs, national regulatory authorities (NRAs) in Egypt, Ghana, Nigeria, South Africa and Tanzania are operating at maturity level 3.^4^ Of these, only NRAs in Egypt and South Africa have vaccine-producing status—like NRAs in China, India and Indonesia. Although manufacturers from these two countries have a competitive advantage, there is an opportunity to expand capacity in other countries with vaccine production projects to achieve required regulatory status to accelerate progress on vaccine manufacturing without undermining regional and global market health.

Evidence suggests that bridging existing regulatory gaps requires a multilevel approach that reflects the need for investment in clinical research capacity.^5^ With efforts under way to operationalize the African Medicines Agency in Rwanda, the Africa Centres for Disease Control and Prevention, the African Medicines Regulatory Harmonization Initiative and the African Vaccine Regulatory Forum are well positioned to work with regional economic communities, GAVI, the RVMC and other partners to facilitate cross-country knowledge transfers to build an integrated clinical trials and regulatory system that supports the sustainable production of health products and immunization progress. The recent commitment by the European Medicines Agency to support the African Medicines Agency is a welcome development.^6^ Although these initiatives may create alternative regulatory pathways for health products, it is vital to ensure alignment with the global benchmark through responsive technical guidance from the WHO.^4^ As multiple donor-funded initiatives coalesce over time, a key measure of progress is that duplication is minimized and regulatory institutions are well-equipped to assess traditional and emerging platform technologies.

Although there are multiple vaccine-related projects in different countries across the continent,^7^ it is more efficient to have a few large-scale manufacturers with sustainable value chains that incorporate input suppliers and product distributors from the continent.^3^ There is a related opportunity for emerging health product manufacturers to focus on essential medicines and routine medical supplies required for immunization and other healthcare interventions. A diffuse network of many African vaccine manufacturers can create unhealthy market dynamics that may undermine the viability of the system in the face of stiff competition from existing cost-efficient large-volume manufacturers from India and China. It is plausible that African manufacturers that can effectively leverage novel platform technologies and manufacturing innovations with context-specific cost savings can achieve sustainable production at scale with a well-trained workforce.^3,8^

The AVMA is therefore a timely instrument in view of multiple steps at the policy and enterprise levels to scale up vaccine production to meet routine and emergency needs.^8^ Afrigen hosts the mRNA technology transfer hub, which aims to develop novel technologies to address health needs in developing countries. The Institut Pasteur de Dakar, Aspen Pharmacare, Biovac, Vacsera, Sothema and Biovaccines Nigeria have partnerships with players in the global vaccines market.^7^ BioNTech has reached key milestones at its site in Rwanda. The African Pharmaceutical Technology Foundation aims to facilitate access to vital technologies and the International Vaccine Institute is investing in crucial cross-country partnerships. The AVMA or a similar tool can advance this progress through pre-milestone and contingent tools that contribute to pandemic preparedness and equitable access to health products.

Although the overdependence of African countries on vaccine imports became salient during the Covid-19 pandemic, there is growing momentum to build a viable vaccine production and delivery infrastructure on the continent. The design of the AVMA should not be perceived as a symbolic decoupling tool to appease the institutional environment of the global vaccines market.^9^ With decoupling, organizations may embrace elements of the institutional environment that advance their legitimacy and enhance their survival.^9^ They may incorporate keywords in their policies and strategies without commensurate changes in their operations and performance assessment. The vaccine inequity witnessed during the Covid-19 pandemic has spurred multiple global health organizations to make public commitments for equity, but the demonstration of these commitments lies in intraorganizational and interorganizational changes that rebuild trust in the global health ecosystem and advance equitable access to health technologies without fragmented projects.^3^

Thus the AVMA should be implemented as a genuine and sustainable demonstration of the commitment of political and technical leaders to support the successful implementation of the Partnerships for African Vaccine Manufacturing (PAVM) approved by African leaders to increase the local production of vaccines to a significant level within the next two decades.^8^ Pharmaceutical industry growth in the Global South shows that achieving sustainable vaccine production is a marathon that requires turning plans and commitments into progressive actions with sustained leadership and partnerships.^10^ The PAVM Framework for Action highlights measurable milestones that can be achieved through the concerted efforts of stakeholders at national, regional, continental and global levels. Despite significant macroeconomic and geopolitical constraints, African leaders must allocate more resources to research to drive the growth of an emerging biopharmaceutical industry, including targeted investments in clinical trials management and harmonized regulatory processes. They must also work towards creating a pooled procurement mechanism that prioritizes African manufacturers, as global health agencies facilitate market access for these African producers.^1,3^

## Data Availability

None.
